# Biosynthesis of Sulfur-Containing tRNA Modifications: A Comparison of Bacterial, Archaeal, and Eukaryotic Pathways

**DOI:** 10.3390/biom7010027

**Published:** 2017-03-11

**Authors:** Mirela Čavužić, Yuchen Liu

**Affiliations:** Department of Biological Sciences, Louisiana State University, Baton Rouge, LA 70803, USA; mtkalc1@lsu.edu

**Keywords:** tRNA modification, sulfur, iron–sulfur cluster, translation

## Abstract

Post-translational tRNA modifications have very broad diversity and are present in all domains of life. They are important for proper tRNA functions. In this review, we emphasize the recent advances on the biosynthesis of sulfur-containing tRNA nucleosides including the 2-thiouridine (s^2^U) derivatives, 4-thiouridine (s^4^U), 2-thiocytidine (s^2^C), and 2-methylthioadenosine (ms^2^A). Their biosynthetic pathways have two major types depending on the requirement of iron–sulfur (Fe–S) clusters. In all cases, the first step in bacteria and eukaryotes is to activate the sulfur atom of free l-cysteine by cysteine desulfurases, generating a persulfide (R-S-SH) group. In some archaea, a cysteine desulfurase is missing. The following steps of the bacterial s^2^U and s^4^U formation are Fe–S cluster independent, and the activated sulfur is transferred by persulfide-carrier proteins. By contrast, the biosynthesis of bacterial s^2^C and ms^2^A require Fe–S cluster dependent enzymes. A recent study shows that the archaeal s^4^U synthetase (ThiI) and the eukaryotic cytosolic 2-thiouridine synthetase (Ncs6) are Fe–S enzymes; this expands the role of Fe–S enzymes in tRNA thiolation to the Archaea and Eukarya domains. The detailed reaction mechanisms of Fe–S cluster depend s^2^U and s^4^U formation await further investigations.

## 1. Introduction

Transfer RNAs (tRNAs) play a crucial role in protein synthesis by serving as a linkage between messenger RNAs (mRNAs) and amino acids. Amino acids are attached to tRNAs during aminoacylation catalyzed by aminoacyl-tRNA synthetases (aaRSs) [1]. The aaRSs define the genetic code by accurately matching cognate tRNAs with their corresponding amino acids. They mischarge tRNA once in ~10^4^ reactions, similar to the error rate of transcription (~10^−4^) and ribosomal decoding (~10^−4^) [1,2]. One reason for these high accuracy levels of aminoacylation and ribosomal decoding reactions is tRNA posttranscriptional modifications that include over 100 different types [3,4]. In order to fullfil their canonical roles in protein synthesis as well as their non-canonical cellular roles, tRNAs are heavily modified to the fully functional states–including removing the 5′-end ladder, splicing introns, adding the 3′-end CCA tail, and numerous post-translational chemical modifications of specific nucleosides [5]. The functions of tRNA posttranscriptional modifications are diverse, such as stabilizing tRNA structures, enabling identification of tRNAs by aaRSs, enhancing ribosomal binding to aminoacylated tRNAs, maintaining reading frame, and ensuring proper codon-anticodon base pairing [6]. While some posttranscriptional modifications are conserved in all domains of life (for example, methylation), other types are specific to one domain (for example, archaeosine in archaea) [7]. In this review, we will focus on sulfur-containing tRNA modifications and recent advances in their biosynthetic pathways.

## 2. Sulfur-Containing Modifications and Their Physiological Roles

**Figure 1:**
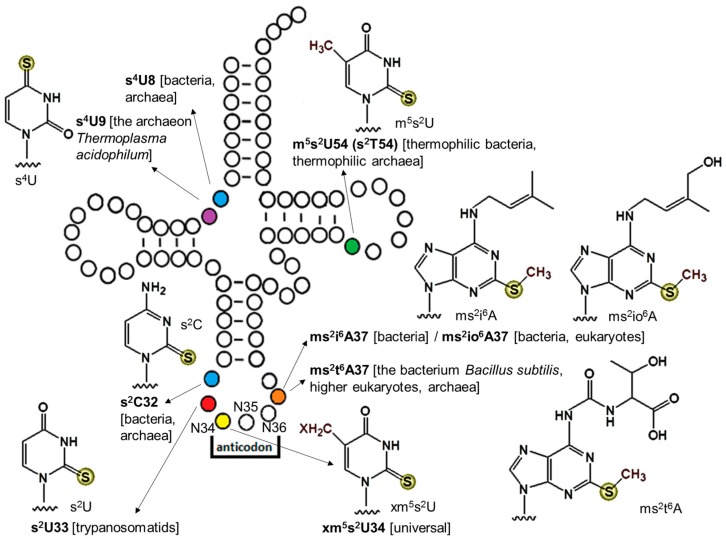
The location and structure of known tRNA thio-modification in three domains of life. The distribution of each modification is indicated in square brackets. Abbreviations: s^4^U, 4-thiouridine; s^2^C, 2-thiocytidine; s^2^U, 2-thiouridine; m^5^s^2^U, 5-methyl-2-thiouridine; s^2^T, 2-thioribothymidine; ms^2^i^6^A, 2-methylthio-*N*^6^-isopentenyladenosine; ms^2^io^6^A, 2-methylthio-*N*^6^-hydroxyisopentenyladenosine; ms^2^t^6^A, 2-methylthio-*N*^6^-threonylcarbamoyladenosine. The xm^5^s^2^U stands for 5-methyl-2-thiouridine derivatives: 5-methylaminomethyl-2-thiouridine (mnm^5^s^2^U), 5-carboxymethylaminomethyl-2-thiouridine (cmnm^5^s^2^U), 5-methoxycarbonylmethyl-2-thiouridine (mcm^5^s^2^U), and 5-taurinomethyl-2-thiouridine (τm^5^s^2^U).

Sulfur-containing modifications are commonly found at seven different tRNA positions: 8, 9, 32, 33, 34, 37, and 54 (Figure 1). These modifications include the 2-thiouridine (s^2^U) derivatives, 4-thiouridine (s^4^U), 2-thiocytidine (s^2^C), and 2-methylthioadenosine (ms^2^A). Only thiolated guanine has not been reported to date. These thio-modifications fulfill versatile functions, and their roles differ according to their positions on tRNAs. Thio-modifications outside the anticodon loop often improve tRNA structural stability, while thio-modifications in the anticodon loop are usually important for translational fidelity and efficiency.

The 4-thiouridine modification at tRNA position 8 (s^4^U8) is conserved in Bacteria and Archaea. It has not yet been reported in eukaryotes, although the gene homolog of the prokaryotic s^4^U8 modification enzyme (ThiI) are present in some eukaryotic genomes [8]. The s^4^U modification has also been reported at position 9 of tRNA^Leu^_UAG_ in the archaeon *Thermoplasma acidophilum* identified by liquid chromatography-tandem mass spectrometry (LC-MS/MS) analysis, along with several other novel modifications [9]. According to the online tRNA database (http://trnadb.bioinf.uni-leipzig.de), s^4^U8 has been found in the virus Enterobacteria phage T4, suggesting that this modification is also present in viruses [10]. Physiologically, s^4^U8 acts as a photosensor of near-ultraviolet (UV) radiation. Following irradiation, U8 cross-links with the structurally nearby cytidine at position 13. This causes tRNA structural changes that prevent tRNA from aminoacylation, and consequently the accumulation of uncharged tRNA triggers stringent cellular responses [11].

The 2-thio (s^2^) modification without any other modifications on the same nucleoside can be found at tRNA positions 32 and 33 as s^2^C32 and s^2^U33, respectively. The s^2^C32 modification is present in *Escherichia coli* and *Salmonella enterica* tRNA^Arg^_ICG_ (decodes CGU/C/A), tRNA^Arg^_CCG_ (decodes CGG), tRNA^Arg^_mnm_^5^_UCU_ (decodes AGA/G), and tRNA^Ser^_GCU_ (decodes AGC/U) [11]; and it has also been found in Archaea [12]. Although the lack of s^2^C32 did not influence the monitored bacterial growth rate, it may affect translation efficiency of rare codons that are intrinsically inefficient in decoding [11,13]. The s^2^U33 modification has been reported in trypanosomatids tRNA^Trp^_CCA_ [14,15]. This modification acts as a negative regulator of the C34 → U34 editing [14]. Without this modification, almost all C34 would be converted to U34, making the UGG codon unreadable.

The s^2^U derivative in the form of 5-methyl-2-thiouridine (m^5^s^2^U), also known as 2-thioribothymidine (s^2^T), at tRNA position 54 has been reported in thermophilic organisms, such as the bacterium *Thermus thermophilus* and the archaeon *Pyrococcus furiosus* [4]. The s^2^ modification of m^5^s^2^U54 raised at elevated temperatures, and the lack of s^2^ led to a temperature sensitive phenotype [4]. Accordingly, m^5^s^2^U54 is proposed to enhance the thermostability of tRNA structures possibly by forming a reverse Hoogsteen base pair with m^1^A58 and stacking with G53 and ψ55 [4].

The U34 located at the first position of the anticodons of tRNA^Gln^_UUG_, tRNA^Lys^_UUU_, and tRNA^Glu^_UUC_ is universally 2-thiolated in all three domains of life. Depending on the organism and the subcellular location, U34 can be hypermodified to different s^2^U derivatives (xm^5^s^2^U) as summarized in Table 1. Several functions have been proposed for the s^2^U34 modification. (i) The rigid conformation of s^2^U―favorably in the C3’-*endo* form [4,16]―at the wobble position leads to a preference for codon:anticodon base paring with A-ending codons [17]. This may be explained by the greater stability of the s^2^U-A vs. s^2^U-G pair [18,19,20]; (ii) The s^2^ group of xm^5^s^2^U acts as an identity element in aminoacylation reactions [21,22,23,24]. In vivo, the mutation of the enzyme (MTO2) responsible for s^2^U34 modification in yeast mitochondria decreased tRNA aminoacylation levels [25]; (iii) The xm^5^s^2^U34 modifications preserve translation fidelity by preventing +1 [26] and +2 [27] ribosome frameshifting; (iv) The s^2^ group of xm^5^s^2^U34 enhances translation efficiency on the ribosome by increasing the binding affinity of aminoacylated tRNAs to the ribosome A-site as well as the GTP hydrolysis rate [21]. Because the s^2^U34 modification has important roles in translation, its absence leads to pleotropic phenotypes in yeast and various diseases in humans. In yeast, the lack of the s^2^U34 modification results in defects in invasive growth [28]; hypersensitivity to high temperatures, rapamycin, caffeine, or oxidative stress [29,30]; inability to maintain normal metabolic cycles [31]; and protein misfolding and aggregation [32]. In humans, impaired s^2^U34 modification of mitochondrial tRNAs has been associated with acute infantile liver failure [33,34] and myoclonic epilepsy with ragged-red fibers [35,36].

The sulfur-containing hypermodified A37, adjacent to the anticodon, is present in tRNAs decoding the UNN codons. In bacteria, either 2-methylthio-*N*^6^-isopentenyladenosine (ms^2^i^6^A) or 2-methylthio-*N*^6^-hydroxyisopentenyladenosine (ms^2^io^6^A) can be found, depending on the presence of the MiaE enzyme responsible for the hydroxylation of i^6^ → io^6^ [11]. The ms^2^i^6^A37 modification is also present in eukaryotes [37,38] and viruses [10]. Additionally, the 2-methylthio-*N*^6^-threonylcarbamoyladenosine (ms^2^t^6^A37) modification has been reported in the bacterium *Bacillus subtilis*, higher eukaryotes [39], and archaea [12]. These modifications are important for the fidelity and efficiency of translation by stabilizing the A-U base paring at the first codon position and preventing +1 frameshift [13]. This is because the A37 modifications bring orders in tRNAs by (i) preventing hydrogen bonding within the anticodon loop and thus ensuring an open loop structure that is required for efficient and correct base paring [18] and (ii) structuring the loop towards the canonical U-turn structure and enhancing the 3’-stack of the codon-anticodon interaction [13]. In bacteria, a ms^2^i^6^ deficiency resulted in a decrease in the polypeptide chain elongation rate leading to a reduced growth rate and a pleiotropic phenotype [11].

## 3. Fe–S Cluster-Dependent and Independent tRNA Thiolation Processes

**Figure 2:**
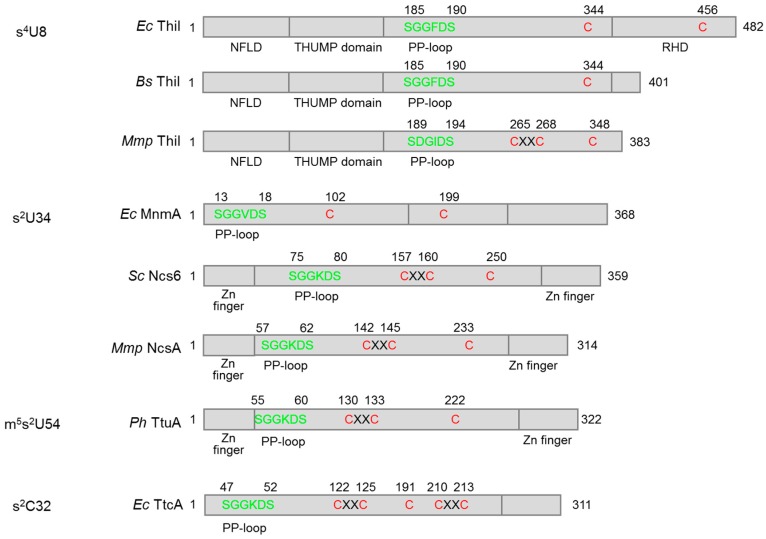
Domain structures of the s^4^U8 synthetase (ThiI), s^2^U34 synthetase (MnmA, Ncs6, or NcsA), m^5^s^2^U54 synthetase (TtuA), and s^2^C32 synthetase (TtcA). The PP-loop (ATP-binding) motif and putative catalytic site Cys residues are colored in green and red, respectively. The domain structures of ThiI, MnmA, and Ncs6 are based on the solved crystal structures of *Bacillus anthracis* ThiI, *E. coli* MnmA, and *Pyrococcus horikoshii* TtuA, respectively. Abbreviations: *Ec*, *E. coli*; *Bs*, *Bacillus subtilis*; *Mmp*, *Methanococcus maripaludis*; *Sc*, *Saccharomyces cerevisiae*; *Ph*, *Pyrococcus horikoshii*; NFLD, N-terminal ferredoxin-like domain; THUMP, thiouridine synthases, methylases and pseudouridine synthases; RHD, rhodanese homology domain.

The biosyntheses of sulfur-containing tRNA modifications usually require multiple enzymes for sulfur transfer. This process generally starts with the activation of sulfur from free l-cysteine by cysteine desulfurases—e.g., IscS in bacteria [40] and Nfs1 in eukaryotes [41]—forming a persulfide (R-S-SH) enzyme adduct and free l-alanine [42]. The persulfidic sulfur, which is covalently linked to a conserved Cys residue of cysteine desulfurase, is then donated via downstream sulfur carrier proteins to the tRNA thiolation enzymes and eventually to tRNA nucleosides. Beside tRNA thiolation, the persulfide on cysteine desulfurase is also the sulfur donor for the biosyntheses of Fe–S clusters and many sulfur-containing vitamins [43]. The tRNA thiolation enzymes are either Fe–S cluster dependent or independent as summarized in Table 2. Domain structures of the s^4^U8, s^2^U34, m^5^s^2^U54, and s^2^C32 synthetases are showed in Figure 2. The biosynthetic pathways of each tRNA thionucleoside are described below.

### 3.1. Biosynthesis of s^4^U8

#### 3.1.1. Bacteria

**Figure 3:**
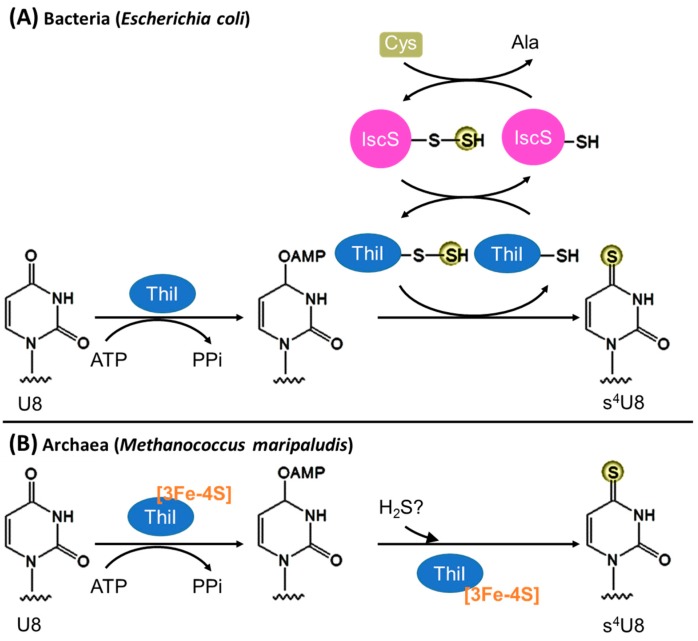
The biosynthetic pathways of tRNA s^4^U8 in Bacteria and Archaea. (**A**) The biosynthetic pathway of s^4^U8 in the bacterium *E. coli* is Fe–S cluster independent, and the sulfur transfer involves persulfide enzyme adducts; (**B**) The biosynthetic pathway of s^4^U8 in the archaeon *M. maripaludis* is Fe–S cluster dependent.

The biosynthesis of s^4^U8 in *E. coli* is Fe–S cluster independent and requires only two proteins—the cysteine desulfurase IscS and the s^4^U8 formation enzyme ThiI (Figure 3A). ThiI contains a PP-loop (ATP-binding) motif and uses ATP to activate the C4 atom of tRNA U8 [60] yielding an adenylated intermediate. For sulfur transfer, IscS first forms a persulfide enzyme adduct using free l-cysteine as the sulfur donor. Then the persulfidic sulfur from IscS is transferred to the first catalytic Cys456 of *E. coli* ThiI, forming a persulfide group on ThiI [44,61]. Subsequently, the second catalytic Cys344 forms a disulfide bond with Cys456 assisting the release of the sulfur from ThiI persulfide [62,63], which is then incorporated into the activated U8 forming s^4^U8. The in vitro reaction requires exogenous reductant (e.g., dithiothreitol) to break the Cys344–Cys456 disulfide bond before the next catalytic round [62], but the physiological reductant is unclear. Notably, the rhodanese homology domain (RHD) that contains the catalytic Cys456, which carries the persulfide, is absent in many bacteria [8,45]; therefore, the sulfur transfer mechanism of RHD-lacking ThiI remains unanswered. The genome of *B. subtilis* encodes four functionally active cysteine desulfurases: SufS, NifZ, NifS, and YrvO [50]. Among them, the *nifZ* gene is adjacent to the *thiI* gene without RHD. Both NifZ and ThiI are essential for s^4^U8 formation in *B. subtilis* [45]; this suggests that the sulfur transfer mechanism of *B. subtilis* ThiI without RHD depends on a specific interaction between NifZ and ThiI.

#### 3.1.2. Archaea

The archaeal s^4^U8 biosynthesis presumably resembles the bacterial ThiI pathway because ThiI is widely distributed in Archaea and the deletion of the *thiI* gene in *Methanococcus maripaludis* results in the elimination of s^4^U in tRNAs [47]. However, the *E. coli* s^4^U biosynthetic mechanism cannot fully explain the archaeal process because the gene encoding a cysteine desulfurase is missing in many sequenced archaeal genomes [8,64] and most archaeal ThiI homologs lack the RHD essential for sulfur transfer. Although the physiological sulfur donor is not known, *M. maripaludis* ThiI can use Na_2_S as an in vitro sulfur donor for tRNA thiolation [47]. The *K*_M_ of Na_2_S is ~1 mM, close to the estimated intracellular concentrations of free sulfide in methanococci (~1–3 mM) [47]; this suggests that sulfide is a physiologically relevant sulfur donor. Furthermore, free l-cysteine is not a sulfur donor for the biosynthesis of Fe–S cluster [64] and tRNA thionucleosides [65] in methanogens; this suggests that a cysteine desulfurase is not required as a central sulfur donor for the biosynthesis of sulfur containing compounds. Notably, the methanogenic archaeal ThiI homologs have three conserved Cys residues (two from a CXXC motif) in the putative catalytic domain [46,47]. A single mutation of any of the three Cys residues abolished *M. maripaludis* ThiI activity [47], implying that all three Cys residues are crucial. Recently, it has been revealed that these three Cys residues coordinate a [3Fe-4S] cluster indispensable for *M. maripaludis* ThiI activity [46]; this indicates that the s^4^U8 biosynthesis in methanogenic Archaea is Fe–S cluster dependent and distinct from the persulfide-based reaction mechanism of bacterial ThiIs (Figure 3B).

### 3.2. Biosynthesis of s^2^U34

#### 3.2.1. Bacteria

**Figure 4:**
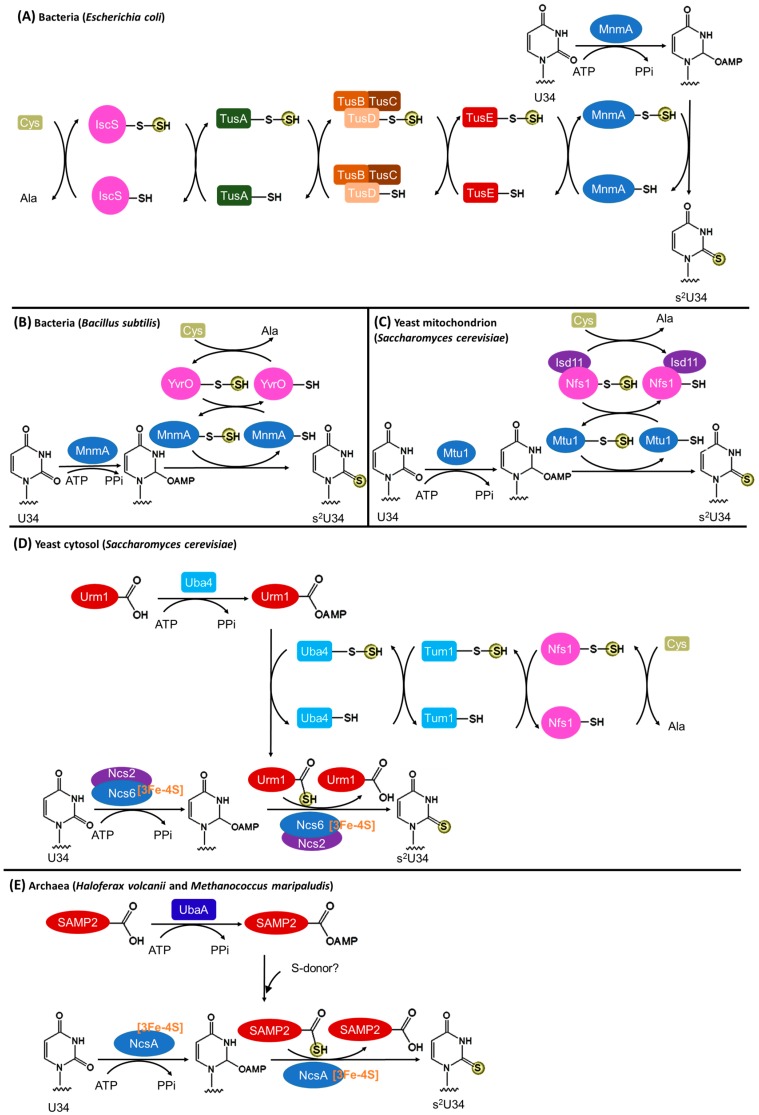
The biosynthetic pathways of tRNA s^2^U34 in all domains of life. (**A**) In *E. coli*, the pathway is Fe–S cluster independent, and the sulfur transfer involves persulfide enzyme adducts; (**B**) *B. subtilis* uses a truncated pathway that is Fe–S cluster independent; (**C**) The yeast mitochondrial pathway may resemble the *B. subtilis* pathway; (**D**) The yeast cytosolic pathway is Fe–S cluster dependent, and the sulfur transfer involves persulfide and thiocarboxylate enzyme adducts; (**E**) The archaeal pathway may resemble the yeast cytosolic pathway.

In bacteria, the biosynthesis of s^2^U34 is Fe–S cluster independent (Figure 4A). In *E. coli*, the persulfide from the cysteine desulfurase IscS is transferred via multiple intermediate sulfur carriers (Tus A, TusBCD complex, and Tus E) in a persulfide-based manner to the s^2^U34 formation enzyme MnmA [66]. Similar to *E. coli* ThiI, MnmA has a PP-loop motif and two active site Cys residues. The PP-loop binds ATP that is consumed to activate the C2 atom of U34 by adenylation. The first catalytic Cys199 receives the sulfur and generates a persulfide enzyme adduct [60]. Then the second catalytic Cys102 forms a disulfide bond with Cys199 facilitating the release of the sulfur from MnmA persulfide, which is finally introduced to the activated U34 forming s^2^U34. Presumably, the Cys344–Cys456 disulfide bond needs to be reduced before the next catalytic round.

Recently, a truncated pathway of s^2^U34 biosynthesis has been revealed in *B. subtilis* [50]. In this bacterium, the intermediate sulfur carries (TusA/BCD/E) are missing (Figure 4B). The cysteine desulfurase YrvO and MnmA are sufficient to introduce s^2^ to U34 [50]; this suggests a direct sulfur transfer from a cysteine desulfurase to the s^2^U34 formation enzyme.

#### 3.2.2. Eukaryotic Mitochondria

The tRNA modification enzyme Mtu1 (mitochondrial tRNA-specific 2-thiouridylase 1) is homologous to bacterial MnmA and responsible for the 2-thiolation reaction of mitochondrial tRNA U34 [48]. Because eukaryotes lack the gene homologs of the intermediate sulfur carriers (TusA/BCD/E) [8], the mitochondrial pathway may resemble the abbreviated *B. subtilis* pathway that requires only a cysteine desulfurase and a 2-thiolation enzyme (Figure 4C). In *Saccharomyces cerevisiae* mitochondria, the cysteine desulfurase Nfs1 forms a complex [67,68] with a small mitochondrial protein Isd11, which is not conserved in prokaryotes. Isd11 is proposed to stabilize Nfs1 in mitochondria.

#### 3.2.3. Eukaryotic Cytosol

The mechanism by which sulfur is incorporated into tRNA s^2^U34 in eukaryotic cytosol differs greatly from that in bacteria (Figure 4D). This process requires the Fe–S cluster assembly machinery [69]. A small amount of the cysteine desulfurase Nfs1 is present in yeast cytosol and participates in tRNA thiolation [70]. The sulfur relay from Nfs1 to the s^2^U34 formation enzyme complex Ncs6/Ncs2 involves several RHD containing proteins and a ubiquitin-like protein. Specifically, from Nfs1, sulfur is transferred to the RHD of Tum1 and then to the RHD of Uba4 as a persulfide group. Uba4 is an E1-like protein that activates Urm1 (ubiquitin-related modifier 1) by adenylation. Then Urm1 receives the sulfur from Uba4, forming a C-terminal thiocarboxylate on Urm1 that may be the proximal sulfur donor for tRNA thiolation [4]. Similar to methanogenic archaeal ThiI, Ncs6 has a PP-loop motif and three conserved Cys residues (two from a CXXC motif) in its putative catalytic domain. The PP-loop binds ATP that is used to adenylate U34, resembling the reaction schemes of ThiI and MnmA. The three Cys residues coordinate a [3Fe-4S] cluster [46], which is probably involved in sulfur transfer. The function of Ncs2 in the Ncs6/Ncs2 complex is still unclear.

#### 3.2.4. Archaea

The archaeal s^2^U34 biosynthetic pathway is proposed to resemble the eukaryotic cytosolic Ncs6 pathway (Figure 4E). This is based on the observations that (i) the *ncs6* gene homologs are widespread in archaeal genomes [8]; (ii) the deletion of the *ncs6* homolog (*ncsA*) in *Haloferax volcanii* resulted in only non-thiolated tRNA^Lys^_UUU_ [71]; (iii) Ncs6 homologs form complexes with the ubiquitin-like small archaeal modifier protein (SAMP), which has high structural homology to Urm1, in *H. volcanii* [72] and in *M. maripaludis* [51]; (iv) the *H. volcanii* E1-like protein UbaA activates SAMP in formation of a thioester intermediate [73]; (v) the deletion of either *samp2* or *ubaA* in *H. volcanii* eliminated thiolated tRNA_Lys_^UUU^ [52]; and (vi) the *M. maripaludis* Ncs6 homolog has a [3Fe-4S] cluster [46]. These findings suggest that both an Fe–S cluster containing Ncs6 homolog and an activated ubiquitin-like protein are required for s^2^U34 formation in Archaea.

### 3.3. Biosynthesis of m^5^s^2^U54

**Figure 5:**
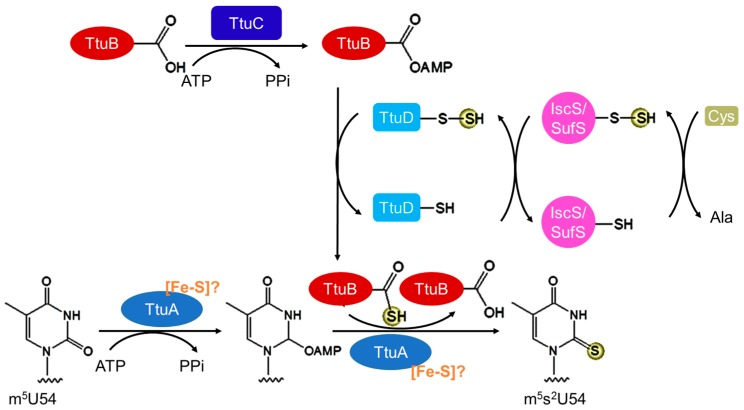
The 2-thiolation process of tRNA U54 in the thermophilic bacterium *T. thermophilus*.

The 2-thiolation process of m^5^s^2^U54 is similar to the Ncs6 pathway in eukaryotic cytosol that requires a RHD containing protein(s), an E1-like enzyme, and an ubiquitin-like protein for sulfur transfer (Figure 5) [4]. The sulfur from free l-cysteine is activated by a cysteine desulfurase (IscS or SufS in *T. thermophilus*) [55,74], which is then transferred as a persulfide group to a recently identified RHD containing protein TtuD [53]. The ubiquitin-like protein TtuB is activated by adenylation catalyzed by an E1-like enzyme TtuC and then receives the activated sulfur, forming a C-terminal thiocarboxylate [54]. TtuA, a homolog of Ncs6, presumably activates m^5^U54 by adenylation and then introduces the sulfur from TtuB thiocarboxylate to tRNA. Similar to Ncs6 and methanogenic archaeal ThiI, three Cys residues (two from a CXXC motif) in the putative catalytic domain of TtuA are important for the thiolation activity [75]. Although a TtuA crystal structure did not reveal the presence of an Fe–S cluster [75], the homology between TtuA and Ncs6 suggests that TtuA may be an Fe–S cluster dependent enzyme.

### 3.4. Biosynthesis of s^2^C32

In bacteria, the biosynthesis of s^2^C32 is dependent on Fe–S cluster formation [76]. For Fe–S cluster assembly, the sulfur from free l-cysteine is transferred via the cysteine desulfurase IscS to IscU, an Fe–S cluster assembly scaffold protein [77]. Fe–S clusters are assembled on IscU and then incorporated into various Fe–S cluster enzymes. The s^2^C formation enzyme TtcA (two-thio-cytidine A), which introduces sulfur to tRNA C32, belongs to the TtcA/TtuA protein family [57]. TtcA has two CXXC motifs, within which three Cys residues coordinate a [4Fe-4S] cluster essential for the thiolation activity [56]. Although the reaction mechanism remains unclear, one Fe atom in the [4Fe-4S] cluster is proposed to transiently ligate a sulfide (–SH) group that is the proximal sulfur donor to generate s^2^C [56].

### 3.5. Biosynthesis of ms^2^A37

The tRNA A37 can be methylthiolated to various ms^2^A37 derivatives. In bacteria, the *N*^6^-isopentenyladenosine (i^6^A37) → 2-methylthio-*N*^6^- isopentenyladenosine (ms^2^i^6^A37) transformation is catalyzed by MiaB, and the *N*^6^-threonylcarbamoyladenosine (t^6^A37) → 2-methylthio-*N*^6^-threonylcarbamoyladenosine (ms^2^t^6^A37) conversion is catalyzed by MtaB, a homolog of MiaB [39]. The human homologs of MiaB and MtaB are CDK5RAP1 (cyclin-dependent-like kinase 5 repressor/activator site-binding protein 1) [58,78] and CDKAL1 (cyclin-dependent-like kinase 5 repressor/activator site-binding protein 1-like 1) [59,79], respectively. In organisms with TcdA, an enzyme that converts t^6^A to a cyclic form ct^6^A, ms^2^ct^6^A37 is formed by MtaB [80]. MiaB/MtaB homologs are also present in archaeal genomes [79], but their functions in methylthiolation have not yet been examined.

Both MiaB and MtaB are methylthiotransferases (MTTases), belonging to the radical *S*-adenosylmethionine (SAM or AdoMet) superfamily of enzymes that catalyze the attachment of a methylthio (-SCH_3_) moiety on unactivated carbon centers. MiaB has two [4Fe-4S] clusters [81,82]. One cluster is coordinated by the characteristic radical SAM motif (CX3CX2C) [81] and is essential for the reductive cleavage of SAM, generating a 5′-deoxyadenosyl radical (Ado^•^) and methionine [83,84]. The second cluster is coordinated by three N-terminal Cys residues and plays a central role in constructing a -SCH_3_ group and attaching it to tRNA [84,85]. The two Fe–S clusters remain intact during catalysis; this indicates that an exogenous sulfur donor possibly attached to the second cluster, instead of the sulfur in Fe–S clusters, is required for this reaction [84].

## 4. Conclusions

The biosynthetic pathways of sulfur-containing tRNA nucleosides are very complex because (i) they usually involve a cascade of sulfur carrier proteins rather than a direct transfer from the ultimate sulfur donor to the substrate; and (ii) the sulfur flows vary significantly between different organisms. Many details (especially in Archaea and eukaryotes) are still waiting to be answered. Just a few examples: (i) because many Archaea lack cysteine desulfurases, the initial sulfur donor for the biosynthesis of thio-modifications in Archaea remains to be identified; (ii) it is unclear whether any unidentified intermediate sulfur carriers are involved in the sulfur transfer between the initial and terminal sulfur transferases for the biosyntheses of s^4^U in Archaea and s^2^U in Archaea and eukaryotes; (iii) the reaction mechanisms of the [4Fe-4S] cluster containing TtcA, the [3Fe-4S] cluster containing methanogenic archaeal ThiI, and the [3Fe-4S] cluster containing archaeal and eukaryotic Ncs6 homologs are still not known; (iv) it remains to be clarified whether TtuA, which is involved in m^5^s^2^U54 biosynthesis in thermophilic prokaryotes, is an Fe–S protein.

## Figures and Tables

**Table 1:** Diversity and distribution of the s^2^U derivatives (xm^5^s^2^U) at position 34 of tRNA^Gln^_UUG_, tRNA^Lys^_UUU_, tRNA^Glu^_UUC_.

xm^5^s^2^U	Name	Distribution
mnm^5^s^2^U	5-methylaminomethyl-2-thiouridine	bacteria, archaea
cmnm^5^s^2^U	5-carboxymethylaminomethyl-2-thiouridine	bacteria, yeast mitochondria
mcm^5^s^2^U	5-methoxycarbonylmethyl-2-thiouridine	eukaryotic cytosol
τm^5^s^2^U	5-taurinomethyl-2-thiouridine	mammalian mitochondria

**Table 2:** The diversity and distribution of tRNA thionucleosides and sulfurtransferases involved in each thiolation.

Nucleoside	Distribution	Model Organisms ^1^	Modification Enzymes (Sulfurtransferases) ^2^	Fe–S Cluster Dependency	Modified tRNA Species	References
s^4^U8	Bacteria	*E. coli*	IscS, ThiI	independent		[44,45]
	Archaea	*M. maripaludis*	S-donor?, ThiI	dependent		[46,47]
s^4^U9	Archaea	*T. acidophilum*	S-donor?, ThiI	independent	tRNA^Leu^_UAG_	[9]
s^4^U33	Eukaryotes	Trypanosomatids	Nfs1/Isd11, Mtu1	independent	tRNA^Trp^_CCA_	[14]
mcm^5^s^2^U34	Eukaryotes	*S. cerevisiae* cytosol	Nfs1, Tum1-RLD, Urm1, Uba4-RLD, Ncs2/Ncs6	dependent	tRNA^Gln, Lys, Glu^	[4]
cmnm^5^s^2^U34	Eukaryotes	*S. cerevisiae* mitohondrion	Nfs1/Isd11, Mtu1	independent	tRNA^Gln, Lys, Glu^	[25]
τm^5^s^2^U34	Eukaryotes	*H. sapiens* mitochondrion	hMTU1	independent	tRNA^Lys^	[48,49]
cmnm^5^s^2^U34/mnm^5^s^2^U34	Bacteria	*E. coli*, *S. enterica*	IscS, TusA, TusBCD, TusE, MnmA	independent	tRNA^Gln, Lys, Glu^	[4]
	Bacteria	*B. subtilis*	YrvO, MnmA	independent	tRNA^Gln, Lys, Glu^	[50]
mnm^5^s^2^U34	Archaea	*H. volcanii*, *M. maripaludis*	S-donor?, SAMP2, UbaA, NcsA	dependent	tRNA^Gln, Lys, Glu^	[46,51,52]
m^5^s^2^U54 (s^2^T54)	Bacteria	*T. thermophilus*	IscS/SufS, TtuA, TtuB, TtuC, TtuD	dependent?		[4,53,54,55]
	Archaea	*P. furiosus*	S-donor?, TtuA, TtuB, TtuC	dependent?		[4]
s^2^C32	Bacteria	*E. coli*	IscS, TtcA	dependent	tRNA^Arg, Ser^	[56,57]
	Archaea		to be determined			
ms^2^i^6^A37/ms^2^io^6^A37	Bacteria	*E. coli*, *S. enterica*	IscS, MiaB	dependent	tRNA^Phe, Tyr, Leu, Ser, Cys, Trp^	[39]
ms^2^i^6^A37	Eukaryotes	*H. sapiens*	CDK5RAP1	dependent	tRNA^Phe, Trp, Tyr^	[58]
ms^2^(c)t^6^A37	Bacteria	*B. subtilis*	IscS, MtaB	dependent	tRNA^Phe, Tyr^	[39]
	Higher eukaryotes	*H. sapiens*	CDKAL1	dependent	tRNA^Ile, Met, Thr, Asn, Lys, Ser, Arg^	[59]
	Archaea		to be determined			

^1^ Abbreviations: *E. coli*, *Escherichia coli*; *M. maripaludis*, *Methanococcus maripaludis*; *T. acidophilum*, *Thermoplasma acidophilum*; *S. cerevisiae*, *Saccharomyces cerevisiae*; *H. sapiens*, *Homo sapiens*; *S. enterica*, *Salmonella enterica*; *B. subtilis*, *Bacillus subtilis*; *H. volcanii*, *Haloferax volcanii*; *P. furiosus*, *Pyrococcus furiosus*. ^2^ The known Fe–S cluster dependent enzymes are highlighted in red. Abbreviations: Isd11, iron sulfur biogenesis desulfurase interacting protein 11; Mtu1, mitochondrial tRNA-specific 2-thiouridylase 1; Tum1, tRNA thiouridine modification protein 1; RLD, rhodanese-like domain; Urm1, ubiquitin-related modifier 1; Uba4, ubiquitin-like protein activator 4; hMTU1, human Mtu1; TusA–E, two-thiouridine synthesis protein A–E; MnmA, 5-methylaminomethyl-2-thiouridine synthetase A; SAMP2, small archaeal modifier protein 2; UbaA, archaeal Uba4 homolog; TtuA-D, two-thiouridine synthesis protein A-D; TtcA, two-thio-cytidine synthetase A; MiaB, N^6^-isopentenyladenosine methylthiotransferase B; CDK5RAP1, cyclin-dependent-like kinase 5 repressor/activator site-binding protein 1; MtaB, N^6^-threonylcarbamoyladenosine methylthiotransferase B; CDKAL1, cyclin-dependent-like kinase 5 repressor/activator site-binding protein 1-like 1.

## References

[1] Ibba M., Söll D. (2000). Aminoacyl-tRNA synthesis. Annu. Rev. Biochem..

[2] Ling J., Reynolds N., Ibba M. (2009). Aminoacyl-tRNA synthesis and translational quality control. Annu. Rev. Microbiol..

[3] Machnicka M.A., Milanowska K., Osman Oglou O., Purta E., Kurkowska M., Olchowik A., Januszewski W., Kalinowski S., Dunin-Horkawicz S., Rother K.M. (2013). MODOMICS: A database of RNA modification pathways—2013 update. Nucleic Acids Res..

[4] Shigi N. (2014). Biosynthesis and functions of sulfur modifications in tRNA. Front. Genet..

[5] Kirchner S., Ignatova Z. (2015). Emerging roles of tRNA in adaptive translation, signalling dynamics and disease. Nat. Rev. Genet..

[6] El Yacoubi B., Bailly M., de Crécy-Lagard V. (2012). Biosynthesis and function of posttranscriptional modifications of transfer RNAs. Annu. Rev. Genet..

[7] Phillips G., de Crécy-Lagard V. (2011). Biosynthesis and function of tRNA modifications in Archaea. Curr. Opin. Microbiol..

[8] Kotera M., Bayashi T., Hattori M., Tokimatsu T., Goto S., Mihara H., Kanehisa M. (2010). Comprehensive genomic analysis of sulfur-relay pathway genes. Genome Inform..

[9] Tomikawa C., Ohira T., Inoue Y., Kawamura T., Yamagishi A., Suzuki T., Hori H. (2013). Distinct tRNA modifications in the thermo-acidophilic archaeon, *Thermoplasma acidophilum*. FEBS Lett..

[10] McClain W.H., Foss K. (1984). Hybrid transfer RNA genes in phage T4. Cell.

[11] Björk G.R., Hagervall T.G. (2014). Transfer RNA modification: Presence, synthesis, and function. EcoSal Plus.

[12] McCloskey J.A., Graham D.E., Zhou S., Crain P.F., Ibba M., Konisky J., Söll D., Olsen G.J. (2001). Post-transcriptional modification in archaeal tRNAs: Identities and phylogenetic relations of nucleotides from mesophilic and hyperthermophilic *Methanococcales*. Nucleic Acids Res..

[13] Gustilo E.M., Vendeix F.A., Agris P.F. (2008). tRNA’s modifications bring order to gene expression. Curr. Opin. Microbiol..

[14] Paris Z., Fleming I.M., Alfonzo J.D. (2012). Determinants of tRNA editing and modification: Avoiding conundrums, affecting function. Semin. Cell Dev. Biol..

[15] Crain P.F., Alfonzo J.D., Rozenski J., Kapushoc S.T., McCloskey J.A., Simpson L. (2002). Modification of the universally unmodified uridine-33 in a mitochondria-imported edited tRNA and the role of the anticodon arm structure on editing efficiency. RNA.

[16] Durant P.C., Bajji A.C., Sundaram M., Kumar R.K., Davis D.R. (2005). Structural effects of hypermodified nucleosides in the *Escherichia coli* and human tRNA^Lys^ anticodon loop: the effect of nucleosides s^2^U, mcm^5^U, mcm^5^s^2^U, mnm^5^s^2^U, t^6^A, and ms^2^t^6^A. Biochemistry.

[17] Alkatib S., Scharff L.B., Rogalski M., Fleischmann T.T., Matthes A., Seeger S., Schöttler M.A., Ruf S., Bock R. (2012). The contributions of wobbling and superwobbling to the reading of the genetic code. PLoS Genet..

[18] Agris P.F., Vendeix F.A., Graham W.D. (2007). tRNA’s wobble decoding of the genome: 40 years of modification. J. Mol. Biol..

[19] Grosjean H., Westhof E. (2016). An integrated, structure- and energy-based view of the genetic code. Nucleic Acids Res..

[20] Shohda K., Okamoto I., Wada T., Seio K., Sekine M. (2000). Synthesis and properties of 2’-*O*-methyl-2-thiouridine and oligoribonucleotides containing 2’-*O*-methyl-2-thiouridine. Bioorg. Med. Chem. Lett..

[21] Rodriguez-Hernandez A., Spears J.L., Gaston K.W., Limbach P.A., Gamper H., Hou Y.M., Kaiser R., Agris P.F., Perona J.J. (2013). Structural and mechanistic basis for enhanced translational efficiency by 2-thiouridine at the tRNA anticodon wobble position. J. Mol. Biol..

[22] Tamura K., Himeno H., Asahara H., Hasegawa T., Shimizu M. (1992). *In vitro* study of *E. coli* tRNA^Arg^ and tRNA^Lys^ identity elements. Nucleic Acids Res..

[23] Sylvers L.A., Rogers K.C., Shimizu M., Ohtsuka E., Söll D. (1993). A 2-thiouridine derivative in tRNA^Glu^ is a positive determinant for aminoacylation by *Escherichia coli* glutamyl-tRNA synthetase. Biochemistry.

[24] Madore E., Florentz C., Giegé R., Sekine S., Yokoyama S., Lapointe J. (1999). Effect of modified nucleotides on *Escherichia coli* tRNA^Glu^ structure and on its aminoacylation by glutamyl-tRNA synthetase. Predominant and distinct roles of the mnm^5^ and s^2^ modifications of U34. Eur. J. Biochem..

[25] Wang X., Yan Q., Guan M.X. (2010). Combination of the loss of cmnm^5^U_34_ with the lack of s^2^U_34_ modifications of tRNA^Lys^, tRNA^Glu^, and tRNA^Gln^ altered mitochondrial biogenesis and respiration. J. Mol. Biol..

[26] Urbonavičius J., Stahl G., Durand J.M., Ben Salem S.N., Qian Q., Farabaugh P.J., Björk G.R. (2003). Transfer RNA modifications that alter +1 frameshifting in general fail to affect −1 frameshifting. RNA.

[27] Brégeon D., Colot V., Radman M., Taddei F. (2001). Translational misreading: A tRNA modification counteracts a +2 ribosomal frameshift. Genes Dev..

[28] Goehring A.S., Rivers D.M., Sprague G.F. (2003). Urmylation: A ubiquitin-like pathway that functions during invasive growth and budding in yeast. Mol. Biol. Cell.

[29] Dewez M., Bauer F., Dieu M., Raes M., Vandenhaute J., Hermand D. (2008). The conserved Wobble uridine tRNA thiolase Ctu1-Ctu2 is required to maintain genome integrity. Proc. Natl. Acad. Sci. USA.

[30] Leidel S., Pedrioli P.G., Bucher T., Brost R., Costanzo M., Schmidt A., Aebersold R., Boone C., Hofmann K., Peter M. (2009). Ubiquitin-related modifier Urm1 acts as a sulphur carrier in thiolation of eukaryotic transfer RNA. Nature.

[31] Laxman S., Sutter B.M., Wu X., Kumar S., Guo X., Trudgian D.C., Mirzaei H., Tu B.P. (2013). Sulfur amino acids regulate translational capacity and metabolic homeostasis through modulation of tRNA thiolation. Cell.

[32] Nedialkova D.D., Leidel S.A. (2015). Optimization of codon translation rates via tRNA modifications maintains proteome integrity. Cell.

[33] Schara U., von Kleist-Retzow J.C., Lainka E., Gerner P., Pyle A., Smith P.M., Lochmüller H., Czermin B., Abicht A., Holinski-Feder E. (2011). Acute liver failure with subsequent cirrhosis as the primary manifestation of *TRMU* mutations. J. Inherit. Metab. Dis..

[34] Zeharia A., Shaag A., Pappo O., Mager-Heckel A.M., Saada A., Beinat M., Karicheva O., Mandel H., Ofek N., Segel R. (2009). Acute infantile liver failure due to mutations in the *TRMU* gene. Am. J. Hum. Genet..

[35] Yasukawa T., Suzuki T., Ishii N., Ohta S., Watanabe K. (2001). Wobble modification defect in tRNA disturbs codon-anticodon interaction in a mitochondrial disease. EMBO J..

[36] Yasukawa T., Suzuki T., Ishii N., Ueda T., Ohta S., Watanabe K. (2000). Defect in modification at the anticodon wobble nucleotide of mitochondrial tRNA^Lys^ with the MERRF encephalomyopathy pathogenic mutation. FEBS Lett..

[37] Suzuki T., Suzuki T. (2014). A complete landscape of post-transcriptional modifications in mammalian mitochondrial tRNAs. Nucleic Acids Res..

[38] Helm M., Alfonzo J.D. (2014). Posttranscriptional RNA modifications: playing metabolic games in a cell’s chemical Legoland. Chem. Biol..

[39] Arragain S., Handelman S.K., Forouhar F., Wei F.Y., Tomizawa K., Hunt J.F., Douki T., Fontecave M., Mulliez E., Atta M. (2010). Identification of eukaryotic and prokaryotic methylthiotransferase for biosynthesis of 2-methylthio-*N*^6^-threonylcarbamoyladenosine in tRNA. J. Biol. Chem..

[40] Mihara H., Esaki N. (2002). Bacterial cysteine desulfurases: Their function and mechanisms. Appl. Microbiol. Biotechnol..

[41] Pandey A., Golla R., Yoon H., Dancis A., Pain D. (2012). Persulfide formation on mitochondrial cysteine desulfurase: Enzyme activation by a eukaryote-specific interacting protein and Fe–S cluster synthesis. Biochem. J..

[42] Kessler D. (2006). Enzymatic activation of sulfur for incorporation into biomolecules in prokaryotes. FEMS Microbiol. Rev..

[43] Black K.A., Dos Santos P.C. (2015). Shared-intermediates in the biosynthesis of thio-cofactors: Mechanism and functions of cysteine desulfurases and sulfur acceptors. Biochim. Biophys. Acta.

[44] Kambampati R., Lauhon C.T. (2000). Evidence for the transfer of sulfane sulfur from IscS to ThiI during the in vitro biosynthesis of 4-thiouridine in *Escherichia coli* tRNA. J. Biol. Chem..

[45] Rajakovich L.J., Tomlinson J., Dos Santos P.C. (2012). Functional analysis of *Bacillus subtilis* genes involved in the biosynthesis of 4-thiouridine in tRNA. J. Bacteriol..

[46] Liu Y., Vinyard D.J., Reesbeck M.E., Suzuki T., Manakongtreecheep K., Holland P.L., Brudvig G.W., Söll D. (2016). A [3Fe-4S] cluster is required for tRNA thiolation in archaea and eukaryotes. Proc. Natl. Acad. Sci. USA.

[47] Liu Y., Zhu X., Nakamura A., Orlando R., Söll D., Whitman W.B. (2012). Biosynthesis of 4-thiouridine in tRNA in the methanogenic archaeon *Methanococcus maripaludis*. J. Biol. Chem..

[48] Umeda N., Suzuki T., Yukawa M., Ohya Y., Shindo H., Watanabe K., Suzuki T. (2005). Mitochondria-specific RNA-modifying enzymes responsible for the biosynthesis of the wobble base in mitochondrial tRNAs. Implications for the molecular pathogenesis of human mitochondrial diseases. J. Biol. Chem..

[49] Suzuki T., Nagao A., Suzuki T. (2011). Human mitochondrial tRNAs: biogenesis, function, structural aspects, and diseases. Annu. Rev. Genet..

[50] Black K.A., Dos Santos P.C. (2015). Abbreviated pathway for biosynthesis of 2-thiouridine in *Bacillus subtilis*. J. Bacteriol..

[51] Liu Y., Long F., Wang L., Söll D., Whitman W.B. (2014). The putative tRNA 2-thiouridine synthetase Ncs6 is an essential sulfur carrier in *Methanococcus maripaludis*. FEBS Lett..

[52] Miranda H.V., Nembhard N., Su D., Hepowit N., Krause D.J., Pritz J.R., Phillips C., Söll D., Maupin-Furlow J.A. (2011). E1- and ubiquitin-like proteins provide a direct link between protein conjugation and sulfur transfer in archaea. Proc. Natl. Acad. Sci. USA.

[53] Shigi N., Asai S.I., Watanabe K. (2016). Identification of a rhodanese-like protein involved in thiouridine biosynthesis in *Thermus thermophilus* tRNA. FEBS Lett..

[54] Shigi N., Sakaguchi Y., Asai S.-I., Suzuki T., Watanabe K. (2008). Common thiolation mechanism in the biosynthesis of tRNA thiouridine and sulphur-containing cofactors. EMBO J..

[55] Shigi N., Suzuki T., Terada T., Shirouzu M., Yokoyama S., Watanabe K. (2006). Temperature-dependent biosynthesis of 2-thioribothymidine of *Thermus thermophilus* tRNA. J. Biol. Chem..

[56] Bouvier D., Labessan N., Clémancey M., Latour J.M., Ravanat J.L., Fontecave M., Atta M. (2014). TtcA a new tRNA-thioltransferase with an Fe–S cluster. Nucleic Acids Res..

[57] Jäger G., Leipuviene R., Pollard M.G., Qian Q., Björk G.R. (2004). The conserved Cys–X1–X2–Cys motif present in the TtcA protein is required for the thiolation of cytidine in position 32 of tRNA from *Salmonella enterica* serovar Typhimurium. J. Bacteriol..

[58] Reiter V., Matschkal D.M., Wagner M., Globisch D., Kneuttinger A.C., Müller M., Carell T. (2012). The CDK5 repressor CDK5RAP1 is a methylthiotransferase acting on nuclear and mitochondrial RNA. Nucleic Acids Res..

[59] Landgraf B.J., McCarthy E.L., Booker S.J. (2016). Radical *S*-adenosylmethionine enzymes in human health and disease. Annu. Rev. Biochem..

[60] Numata T., Ikeuchi Y., Fukai S., Suzuki T., Nureki O. (2006). Snapshots of tRNA sulphuration via an adenylated intermediate. Nature.

[61] Palenchar P.M., Buck C.J., Cheng H., Larson T.J., Mueller E.G. (2000). Evidence that ThiI, an enzyme shared between thiamin and 4-thiouridine biosynthesis, may be a sulfurtransferase that proceeds through a persulfide intermediate. J. Biol. Chem..

[62] Mueller E.G., Palenchar P.M., Buck C.J. (2001). The role of the cysteine residues of ThiI in the generation of 4-thiouridine in tRNA. J. Biol. Chem..

[63] Veerareddygari G.R., Klusman T.C., Mueller E.G. (2016). Characterization of the catalytic disulfide bond in *E. coli* 4-thiouridine synthetase to elucidate its functional quaternary structure. Protein Sci..

[64] Liu Y., Sieprawska-Lupa M., Whitman W.B., White R.H. (2010). Cysteine is not the sulfur source for iron-sulfur cluster and methionine biosynthesis in the methanogenic archaeon *Methanococcus maripaludis*. J. Biol. Chem..

[65] Rauch B.J., Perona J.J. (2016). Efficient sulfide assimilation in *Methanosarcina acetivorans* is mediated by the MA1715 Protein. J. Bacteriol..

[66] Ikeuchi Y., Shigi N., Kato J., Nishimura A., Suzuki T. (2006). Mechanistic insights into sulfur relay by multiple sulfur mediators involved in thiouridine biosynthesis at tRNA wobble positions. Mol. Cell.

[67] Adam A.C., Bornhövd C., Prokisch H., Neupert W., Hell K. (2006). The Nfs1 interacting protein Isd11 has an essential role in Fe/S cluster biogenesis in mitochondria. EMBO J..

[68] Wiedemann N., Urzica E., Guiard B., Müller H., Lohaus C., Meyer H.E., Ryan M.T., Meisinger C., Mühlenhoff U., Lill R. (2006). Essential role of Isd11 in mitochondrial iron-sulfur cluster synthesis on Isu scaffold proteins. EMBO J..

[69] Nakai Y., Nakai M., Lill R., Suzuki T., Hayashi H. (2007). Thio modification of yeast cytosolic tRNA is an iron-sulfur protein-dependent pathway. Mol. Cell. Biol.

[70] Mühlenhoff U., Balk J., Richhardt N., Kaiser J.T., Sipos K., Kispal G., Lill R. (2004). Functional characterization of the eukaryotic cysteine desulfurase Nfs1p from *Saccharomyces cerevisiae*. J. Biol. Chem..

[71] Chavarria N.E., Hwang S., Cao S., Fu X., Holman M., Elbanna D., Rodriguez S., Arrington D., Englert M., Uthandi S. (2014). Archaeal Tuc1/Ncs6 homolog required for wobble uridine tRNA thiolation is associated with ubiquitin-proteasome, translation, and RNA processing system homologs. PLoS ONE.

[72] Humbard M.A., Miranda H.V., Lim J.M., Krause D.J., Pritz J.R., Zhou G., Chen S., Wells L., Maupin-Furlow J.A. (2010). Ubiquitin-like small archaeal modifier proteins (SAMPs) in *Haloferax volcanii*. Nature.

[73] Hepowit N.L., de Vera I.M., Cao S., Fu X., Wu Y., Uthandi S., Chavarria N.E., Englert M., Su D., Söll D. (2016). Mechanistic insight into protein modification and sulfur mobilization activities of noncanonical E1 and associated ubiquitin-like proteins of Archaea. FEBS J..

[74] Shigi N., Sakaguchi Y., Suzuki T., Watanabe K. (2006). Identification of two tRNA thiolation genes required for cell growth at extremely high temperatures. J. Biol. Chem..

[75] Chen M., Narai S., Omura N., Shigi N., Chimnaronk S., Tanaka Y., Yao M. (2016). Crystallographic study of the 2-thioribothymidine-synthetic complex TtuA-TtuB from *Thermus thermophilus*. Acta Crystallogr. F Struct. Biol. Commun..

[76] Leipuviene R., Qian Q., Björk G.R. (2004). Formation of thiolated nucleosides present in tRNA from *Salmonella enterica* serovar Typhimurium occurs in two principally distinct pathways. J. Bacteriol..

[77] Mueller E.G. (2006). Trafficking in persulfides: Delivering sulfur in biosynthetic pathways. Nat. Chem. Biol..

[78] Wei F.Y., Zhou B., Suzuki T., Miyata K., Ujihara Y., Horiguchi H., Takahashi N., Xie P., Michiue H., Fujimura A. (2015). Cdk5rap1-mediated 2-methylthio modification of mitochondrial tRNAs governs protein translation and contributes to myopathy in mice and humans. Cell Metab..

[79] Kaminska K.H., Baraniak U., Boniecki M., Nowaczyk K., Czerwoniec A., Bujnicki J.M. (2008). Structural bioinformatics analysis of enzymes involved in the biosynthesis pathway of the hypermodified nucleoside ms^2^io^6^A37 in tRNA. Proteins.

[80] Kang B.I., Miyauchi K., Matuszewski M., D’Almeida G.S., Rubio M.A., Alfonzo J.D., Inoue K., Sakaguchi Y., Suzuki T., Sochacka E. (2017). Identification of 2-methylthio cyclic *N*^6^-threonylcarbamoyladenosine (ms^2^ct^6^A) as a novel RNA modification at position 37 of tRNAs. Nucleic Acids Res..

[81] Hernández H.L., Pierrel F., Elleingand E., García-Serres R., Huynh B.H., Johnson M.K., Fontecave M., Atta M. (2007). MiaB, a bifunctional radical-*S*-adenosylmethionine enzyme involved in the thiolation and methylation of tRNA, contains two essential [4Fe-4S] clusters. Biochemistry.

[82] Pierrel F., Björk G.R., Fontecave M., Atta M. (2002). Enzymatic modification of tRNAs: MiaB is an iron-sulfur protein. J. Biol. Chem..

[83] Pierrel F., Douki T., Fontecave M., Atta M. (2004). MiaB protein is a bifunctional radical-*S*-adenosylmethionine enzyme involved in thiolation and methylation of tRNA. J. Biol. Chem..

[84] Forouhar F., Arragain S., Atta M., Gambarelli S., Mouesca J.M., Hussain M., Xiao R., Kieffer-Jaquinod S., Seetharaman J., Acton T.B. (2013). Two Fe–S clusters catalyze sulfur insertion by radical-SAM methylthiotransferases. Nat. Chem. Biol..

[85] Landgraf B.J., Arcinas A.J., Lee K.H., Booker S.J. (2013). Identification of an intermediate methyl carrier in the radical *S*-adenosylmethionine methylthiotransferases RimO and MiaB. J. Am. Chem. Soc..

